# Exposure of health workers in primary health care to glutaraldehyde

**DOI:** 10.1186/1745-6673-8-31

**Published:** 2013-11-01

**Authors:** M Angel González Jara, Alfonso Mora Hidalgo, J Carlos Avalos Gulin, Marcos López Albiach, Laura Muñoz Ortiz, Pere Torán Monserrat, Xavier Esteva Ollé

**Affiliations:** 1 Basic Unit of Prevention Metropolitana Nord, Catalan Health Institute, Carrer Torrent de Can Gaio 17, 08320 El Masnou, Spain; 2 Central Unit of Prevention, Catalan Health Institute, Av. Gran Via de les Corts Catalanes 587, 08007, Barcelona, Spain; 3 Research Support Unit Metropolitana Nord, IDIAP Jordi Gol and Catalan Health Institute, Carrer Major 49-53 (1st floor), 08921, Santa Coloma de Gramenet, Spain

## Abstract

**Background:**

In order to avoid proliferation of microorganisms, cleaning, disinfection and sterilisation in health centres is of utmost importance hence reducing exposure of workers to biological agents and of clients that attend these health centres to potential infections. One of the most commonly-used chemical is glutaraldehyde. The effects of its exposure are well known in the hospital setting; however there is very little information available with regards to the primary health care domain.

**Objective:**

To determine and measure the exposure of health workers in Primary Health Care Centres. Environmental to glutaraldehyde and staff concentration will be measured and compared with regulated Occupational Exposure Limits.

**Methods/Design:**

Observational, cross-sectional and multi-centre study. The study population will be composed of any health professionals in contact with the chemical substance that work in the Primary Health Care Centres in the areas of Barcelonès Nord, Maresme, and Barcelona city belonging to the Catalan Institute of Health.

Data will be collected from 1) Glutaraldhyde consumption from the previous 4 years in the health centres under study. 2) Semi-structured interviews and key informants to gather information related to glutaraldehyde exposure. 3) Sampling of the substance in the processes considered to be high exposure.

**Discussion:**

Although glutaraldehyde is extensively used in health centres, scientific literature only deals with certain occupational hazards in the hospital setting.

This study attempts to take an in-depth look into the risk factors and environmental conditions that exist in the primary care workplace with exposure to glutaraldehyde.

## Background

Cleaning, disinfection and sterilisation are the main tools used to avoid microorganism proliferation and therefore infection [1]. This has been a challenge for managers as the nosocomial infections significantly increase costs, jeopardise the quality of care and endanger the health and safety of patients and workers.

These processes require the use of chemicals (detergents and disinfectants) at all times, which must be inevitably used on certain occasions [2,3]. However, these chemicals are not always used properly: disinfection prior to sterilisation is a common practice that conflicts with official recommendations [4]. This practice is unnecessary and only causes an increase in the economic costs and increased exposure to such substances.

In this regard, the fact that no established structured training has been carried out that will increase the level of knowledge and skills of health professionals regarding these activities is very striking. In the majority of cases, learning is based on the informal transmission of knowledge, skills and attitudes from one set of professionals to another. While protocols on cleaning, disinfection and sterilisation are in place in many centres, its dissemination and implementation is poor, where these are often not updated and many of the workers involved in these tasks are in fact unaware of their existence [5-8].

Glutaraldehyde is the chemical disinfectant mainly used in primary care centres. It is a high-level disinfectant (HLD) classified as toxic and sensitizing. The threshold limit value (TLV) that has been recommended since 1998 by the American Conference Industrial Hygienists (ACGIH) for glutaraldehyde is 0.05 ppm as a ceiling value (TLV-C), with the following scores: sen (sensitizing substance for skin contact or inhalation) and A4 (not classifiable as a human carcinogen).

The potential effects that exposure may cause are well known: the onset of asthma in exposed workers [9], contact dermatitis [10] and/or irritating effects to the respiratory tract and skin [11]. There is no current evidence of carcinogenic activity of glutaraldehyde owing to its exposure [12].

Those health workers exposed to glutaraldehyde are nurses from endoscopy units and surgical theatre [13], Xray technicians [14], odontologists [15] and lab technicians [16].

Factors that contribute to exposure of glutaraldehyde are inadequate and unsafe working practices [17], insufficient ventilation [18], and the failure to use goggles, protective clothing and gloves [19].

The air concentration of a chemical substance is used to quantitatively assess the level of exposure to workers. There is scientific evidence that shows glutaraldehyde air concentration in the workplace, namely hospitals: measurements in the endoscopy department showed a mean concentration of 3,7 ± 7,4 mg/m^3^[20]; in surgical theatres values above the limit of 0.05 ppm [17]; or in the Xray services an average of 0.0018 mg/m^3^, levels in the darkroom were five times over the legal limit values [21].

We believe that the importance of this study lies in the scarcity of scientific publications that objectively assess the exposure of health professionals in primary health care to glutaraldehyde. We hope the results will provide scientific evidence that currently does not exist on the exposure to this substance in primary care centres, in which a major percentage of health workers work.

The main objective of this project is to assess the exposure of health professionals in the Primary Health Care sector through the measurement of glutaraldehyde air concentration and staff concentration and to compare them to permissible exposure limits (PEL).

The secondary objective is to possess information about exposure: physical location of the sites where the disinfectant is being handled, environmental conditions of these areas, times of greatest exposure, and professional workers exposed.

## Methods/Design

Observational, cross-sectional and multi-centric study. The population of the study will be any health professionals in contact with the chemical substance that work in the Primary Health Centres in the areas of Barcelonès Nord and Maresme, and Barcelona city belonging to the Catalán Institue of Health.

The study covers six Primary Care Services (SAP Badalona-Sant Adrià de Besòs, SAP Santa Coloma de Gramenet, SAP Mataró- Maresme, SAP Dreta, SAP Esquerra, SAP Litoral y SAP Muntanya), with a total of 85 health centres with more than 7,000 employees.

Out of the entire staff, only non-medical health personnel (nurses and nursing assistants) and medical staff (doctors and dentists) who have contact with it (directly or indirectly) during the workday shall be considered as exposed personnel. The study population is initially estimated to comprise 6,420 workers. The exact number of employees who will participate in measurements cannot be confirmed yet due to the fact that this depends on the data obtained in the hygienic survey in order to perform the sampling strategy.

Table 1 shows the primary and secondary variables to be collected throughout the study.

**Table 1:** Main and secondary variables


**Primary variables**	
Workers concentration of glutaraldehyde (mg/m^3^)	
Environment concentration of glutaraldehyde (mg/m^3^)	
**Secondary variables**	
**Staff**	Professional category
	Gender
	Age
	Professional experience (in years)
	Working shift (morning, afternoon, part-time)
**Process**	Maximum exposure to glutaraldehyde process
	Weekly exposure time (in minutes)
	Individual protection equipment during handling
	Product type (chemical substance)
**Structure**	Premises, hall or consultation room with glutaraldehyde
	Premises size (m^3^)
	Premises temperature °C
	Premises relative humidity (%)
	Premises CO2 level (ppm)
	Type of ventilation (natural or artificial)
**Procedure**	Existence of cleaning, disinfecting and sterilization protocols (Y/N)
	Existence of glutaraldehyde handling protocols (Y/N)
	Training of workers (Y/N)
	Kind of information provided to the workers

### Data collection

The study has 3 stages with different source of information.

Phase 1. Glutaraldehyde consumption in the health centres included in the study over the previous 4 years. The Economy and Financial department will be in charge of providing the information regarding quantities acquired and volumes utilized, expressed in litres, in the different health centres. The strategy to select the health workers will be established from these results.

Phase 2. Semi-structured interviews and key informants (management teams and professionals who handle the product) to gather information related to glutaraldehyde exposure. A Hygienic Survey is the industrial hygiene procedure which aims to obtain all the information needed to make a judgment on issues related to chemical exposure. This process has the objective of collecting data not only related to the product but also to the following aspects: substance inventory, process flow chart, exposed worker position and professional categories, exposure time, rooms where glutaraldehyde is present, environmental conditions where the product is used, worker information on the kind of products they use, product safety cards, safety measures to handle the product and workers training. This information will lead to the strategy of sampling the product.

Phase 3. Sampling of the substance in the environment and in the staff that will provide the study variables. The initial estimate is the performance of about 178 hygenic determinations.

### Measurement strategy to determine glutaraldehyde

The sampling strategy for personal and environmental assessments will take into account all areas where there is a potential contaminant source.

Samples will be taken from all rooms where glutaraldehyde is handled: odontology consultation rooms, medical material cleaning rooms, emergency boxes, sterilisation room.

**Figure 1:**
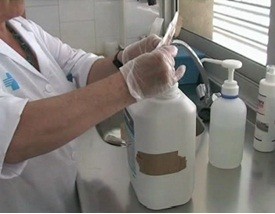
Activation of the substance and filling of the tray.

The timeframe is determined by the moment when the substance is at maximum exposure, such as:

•The time taken to activate the substance and fill the tray (Figure 1).

**Figure 2:**
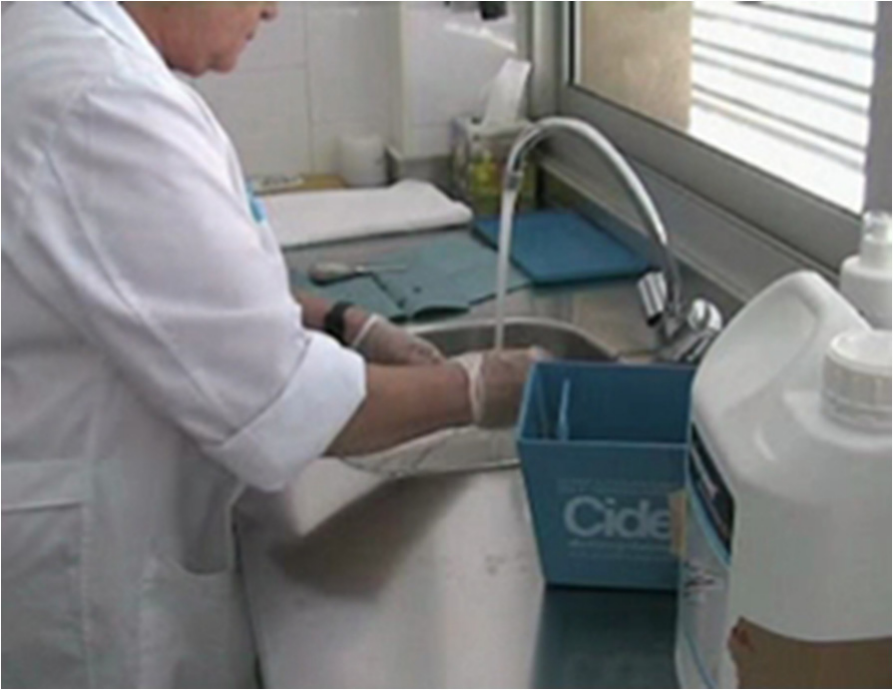
Cleaning medical material next to the tray and dipping it into the glutaraldehyde tray.

•The time taken to clean medical material next to the tray and dip it into the glutaraldehyde tray (Figure 2).

**Figure 3:**
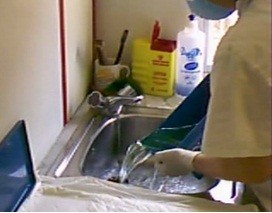
Emptying the glutaraldehyde from the tray and cleaning it.

•The time taken to empty the glutaraldehyde from the tray and clean the tray (Figure 3).

**Figure 4:**
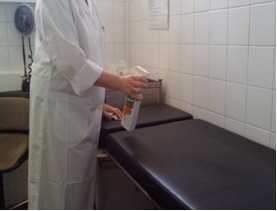
Cleaning and disinfecting surfaces.

•The time taken to clean and disinfect surfaces (Figure 4).

The measurements of the staff will take 15 minutes and 60 minutes for the environment.

The workers with higher levels of exposure to glutaraldehyde will be attached to an aspiration pump that will determine their exposure level.

### Operational procedure to determine exposure level

Sampling and analysis will be performed by the Instituto Nacional de Seguridad e Higiene en el Trabajo (INSHT): “Glurataldehyde determination in the air through adsorption method using silica gel coated with 2,4 dinitrophenylhydrazine and High Performance Liquid Chromatography with UV detection” is an active sampling by solid adsorbent (silica gel).

Before and after each measurement, the pump will be calibrated through the primary airflow meter DryCal® DC_Lite Bios obtaining ten readings for previous and 10 for subsequent flows for each measurement. To calculate the concentration, average flow will be used.

To transport and preserve the samples for the purpose of avoiding contamination, they will be kept inside a small box in a fridge. A control tube (white) will be inserted for each batch.

To determine the exposure levels, the collection equipment will be first attached to the exposed professional for 15 minutes. Afterwards, several collection devices will be placed near the contamination focus for a minimum of 60 minutes.

During the determination of exposure levels, other data will be collected: air temperature, relative humidity, CO2 levels, room area, room air renovation, data regarding handling of the chemical substance and the number of exposed workers.

Measurement instruments and Material:

•Four low flow sampling pumps SKC®, model Universal DE Luxe, Eex ia IIC T4.

•Silica gel SKC Ref. 226–119 (silica gel coated with 2.4 DNPH).

•Primary calibrator DryCal® DC-Lite Bios, model DCL-M.

•Therrmo-hygrometer model TES 1360.

•Ambient CO_2_ level meter TESTO 535.

Additional material: measurement instruments, silicone tubs (1.2 cm in external diameter and 0.6 in internal) and teflon.

### Analysis

Microsoft Office Access and Stata version 11 will be used to manage the data. Once the data have been entered, the quality of the data will be evaluated, treating extreme, incongruent and rare values.

For the main objective, there will be a descriptive analysis of the global concentration of glutaraldehyde both in the environment and in the staff and this will be compared with legal limits.

For the secondary objectives, a descriptive analysis will be performed of the product concentration stratified by room, employment position, professional categories and for tasks identified for maximum exposure. The association of glutaraldehyde concentration and CO2 levels, temperature, relative humidity, ventilation and room area will also be studied. For the association of quantitative variables, the Pearson correlation coefficient will be used and for qualitative variables, Student’s t-test will be used. All statistical tests will have a confidence interval of 95%.

## Discussion

The use of chemical substances during cleaning, disinfection and sterilisation are unavoidable: detergents to clean, disinfectants for term-sensitive materials and equipment and working surfaces, and high performance disinfectants to sterilise medical material [7,22]. The excellent properties of glutaraldehyde makes it the first choice in health centres in terms of considerations for health and safety in the workplace. Many papers illustrated and described the hazards of glutaraldehyde to the exposed health workers in the hospital environment [17,18]. However there is very little evidence in the primary care setting even though it is more utilized by the population.

The lack of studies on the use and exposure to these types of substances of workers in primary health care is paradoxical when, in a health district such as ours, the proportion of health workers in primary care settings doubles the number of workers in hospital care. This situation highlights that there may be large groups of workers likely to be subjected to unstudied exposures, also generating situations of inequality in terms of hygiene and safety in the workplace for workers in the same group.

This study intends to evidence the utilization of glutaraldehyde in the primary health care centres and the hazard exposure to its health workers. At the same time, it aims to provide evidence or data to help us to find out more accurately aspects related to exposure to the chemical disinfectant in the primary care setting.

One limitation of the study lies in the knowledge of the population susceptible to exposure in order to better define the sampling strategy. For this reason, the starting point is those workers who by virtue of their roles may be exposed at some point in their work (nurses, doctors and medical assistants). Because of the nature and organisation of primary care, this ratio is high in our case (about 90%), and we consequently expect that surveys performed in Phase 2 of the study will help establish better the population susceptible to exposure.

The sampling strategy established in this study involves making staff and environmental hygienic determinations at all possible periods identified a priori as maximum exposure and at all areas where there are trays with the substance (an estimation of 178 determinations). The full sample periods which presumably give the most unfavourable conditions minimises the constraints imposed by having to randomly select 15-minute periods out of the entire workday, even if this means carrying out a large number of measurements.

The probability of exceeding the TLV-C value in any of the periods not sampled will need to be subsequently calculated based on the results.

The results of this study will lead to a revision of the cleaning, disinfecting and sterilisation processes and protocols in primary health care [2,3,5]. Moreover, information about these processes of handling hazardous substances, use of security cards, existence and implementation of protocols and use of protective equipment will be provided by the study.

Other studies have shown the need to deepen the awareness of primary care professionals in order to possess knowledge and to adopt evidence-based practices and standard operating procedures or practice guidelines [23,24]. In this sense, we believe that the results of the study may be useful for those systems with a decentralised primary care network in their hospital system. We believe that the data provided by the study can be generalised in such health systems, particularly in relation to defining job posts and the group of primary health care workers likely to be exposed to these substances.

In short, the situation analysis will allow to compare diferent health centres and to propose improvements to protective measures or, eventually, to propose another less harmful chemical instead of gluraldehyde. If glutaraldehyde can not be replaced, preventive actions to reduce exposure to vapors of this substance will be proposed.

It is therefore necessary to establish safe environment workplaces wherever glutaraldehyde is used [25]. Precautions to be taken during handling in order to avoid breathing in vapours are as follows: the introduction of automatic washing machines [5] has been proposed; that it must not be used in the form of a spray or aerosol; that it is not handled or emptied in the presence of flammable vapour; that goggles, protective clothing and gloves are worn; that everyone washes thoroughly with soap and water after handling, that contaminated clothing are removed and washed before reuse, that containers are kept closed and that adequate ventilation is used [26]. Training and information to the exposed workers also reduces the exposure to glutaraldehyde [27]. On the other hand, the results of the study will serve as a starting point for assessing the effectiveness of interventions that may be implemented in order to reduce exposures that may endanger the health of workers [28].

While it is not the aim of the study to identify and describe the health effects of the exposed health workers, it will provide the knowledge about the number of primary healthcare workers at risk and their professional category that may allow to plan future protective measures and perhaps to investigate low concentration chronic exposure.

The project is currently at the end of the field work and data collection stage. Preliminary results will be available in a few months.

### Ethical considerations

All workers involved will be informed of the objectives of the study. This study has been approved by the Ethical Committee of Clinical Investigation of the Primary Care Research Institute Jordi Gol (Barcelona, Spain).

## Abbreviations

ACGIH: American conference industrial hygienists; DNPH: Dinitrophenylhydrazine; HLD: High-level disinfection; ICS: Institut Català de la Salut (Catalan Institute of Health); INSHT: Instituto Nacional de Seguridad e Higiene en el Trabajo (National Institute of Safety and Hygiene at Work, Spain); OEL: Occupational exposure limits; OPA: Orto-ftalaldehyde; PEL: Permissible exposure limit; PHCC: Primary health care centres; STELs: Short term exposure limit; SAP: Servicio de Atención Primaria (Primary Healthcare Service); TLV: Threshold limit value; TLV-C: Threshold limit value ceiling.

## Competing interests

The author’s state that they have no competing interests.

## Authors’ contributions

MAGJ contributed to the original research idea about glutaraldehyde exposure in primary health care and prepared the first draft of the manuscript. MAGJ, JCAG, AMH, MLA, PTM and LMO participated in the design of the research protocol. LMO and PTM provided scientific support and a methodological expert review of the manuscript. All the authors have read, revised and approved the final manuscript.

## Authors’ information

MAGJ, AMH, JCAG y MLA work as high level technicians in the preventive service of ICS.
